# Maternal Anxiety Symptoms and Chinese Adolescents' Mental Health During the COVID-19 Pandemic: The Protective Role of Adolescents' Self-Compassion

**DOI:** 10.3389/fpsyt.2022.837846

**Published:** 2022-04-08

**Authors:** Tong Zhou, Xiaohua Bian, Kening Zhang, Shanyun Zheng, Yinuo Lin, Hong Zheng, Junsheng Liu, Julia Finan

**Affiliations:** ^1^Shanghai Key Laboratory of Mental Health and Psychological Crisis Intervention, Affiliated Mental Health Center (ECNU), School of Psychology and Cognitive Science, East China Normal University, Shanghai, China; ^2^School of Educational Science, Zhengzhou Normal University, Zhengzhou, China; ^3^Department of Counseling, Higher Education, and Special Education, University of Maryland-College Park, College Park, MD, United States; ^4^Shanghai Changning Mental Health Center, Shanghai, China; ^5^Maxwell School of Citizenship and Public Affairs, Syracuse University, Syracuse, NY, United States

**Keywords:** maternal anxiety, psychological maladjustment, self-compassion, COVID-19, Chinese adolescents

## Abstract

The COVID-19 outbreak triggered dramatic changes to family life. Parents, especially mothers, were found to experience more psychological distress during the pandemic, which may have had an impact on their children's mental health. The primary goal of this study was to examine the potential protective role of adolescents' self-compassion in the relationship between maternal anxiety and adolescents' mental health during the COVID-19 pandemic. Participants included 5,720 adolescents (48.9% girls; *M*_age_ = 11.60, *SD*_age_ = 1.36) and their mothers from Zhengzhou city, Henan province, in Mainland China. Adolescents reported their level of self-compassion, PTSD symptoms, and negative affect during the COVID-19 pandemic. Mothers reported their own anxiety symptoms and their children's depression and anxiety symptoms. Results indicated that older female adolescents reported higher levels of PTSD symptoms and negative affect and lower levels of self-compassion than their counterparts. Maternal anxiety during the COVID-19 pandemic was consistently positively associated with adolescents' psychological maladjustment. These associations were buffered by adolescents' self-compassion. Specifically, adolescents with higher levels of self-compassion were found to be less psychologically affected by their mothers' anxiety during the COVID-19 pandemic. Findings highlighted the possibility of improving adolescents' mental health through fostering their self-compassion during the COVID-19 pandemic.

## Introduction

The outbreak of the 2019 coronavirus disease (COVID-19) and its public health responses, such as stay-at-home policies and school closures, triggered “a complex worldwide stressor” (1). In China, empirical studies found that around 30–40% of adolescents were prone to depressive and anxiety symptoms (2), and around 15% of adolescents showed post-traumatic stress disorder (PTSD) symptoms (3) after the COVID-19 outbreak. In the family environment, the pandemic outbreak brought more substantial changes in family routines, which would undoubtedly affect many aspects of family members' lives and may further exacerbate their mental health condition. As such, researchers have made urgent calls for paying more attention to mental health problems in both parents and children (4, 5). Studies found that mothers were likely to perceive more health risk (6) and experience more psychological distress, such as anxiety symptoms, due to the COVID-19 outbreak (7). The transactional process of psychological distress (i.e., anxiety) from parent to child has also been widely documented in previous studies (8–10). However, few empirical studies have explored such issues in the context of COVID-19 with limited research probing the possible protective factors that may buffer the effects of maternal anxiety on children's mental health. Thus, this study aimed to examine the link between maternal anxiety and adolescents' psychological maladjustment (i.e., PTSD symptoms, negative affect, depression, and anxiety) in the family environment as well as the potential protective role of adolescents' self-compassion that may buffer the impact of maternal anxiety during the COVID-19 pandemic.

Anxiety refers to an emotional response to an assessment of uncertainty and potential threat (11). The outbreak of the COVID-19 pandemic imposed a great deal of uncertainty and threat in the family environment, including fear of infection, challenges of homeschooling, disrupted family routines, and lack of social interaction with others (12). As adolescents naturally have an underdeveloped self-regulatory system (13) and as peer interactions are their primary source of social activity (14), it is possible that COVID-19–related stress would exacerbate the emotional challenges adolescents typically face, increasing the risk for poor mental health (15–17). Parents also experienced challenges during the COVID-19 pandemic, including financial stress, which may result in anxiety symptoms. For example, research in Canada found that 35.7% of parents reported being extremely or very anxious about COVID-19 (18). Another study in the United States found that the challenges of caregiving during the COVID-19 pandemic could lead parents to experience anxiety and depressive symptoms (19). In China, during the COVID-19 pandemic, the detection rate of mild anxiety symptoms in parents was 20.7% while that of moderate-to-severe anxiety symptoms was 4% in parents, where parents with school-aged children (ages 5–17) reported more anxiety symptoms (20).

According to the spillover hypothesis (21), affect and behavior from one environment or connection can transfer to another within the family system. Parental negative affect during the COVID-19 pandemic, such as anxiety symptoms, can be directly linked to children's negative emotional outcomes in the home. The literature on COVID-19 supports the spillover hypothesis. A longitudinal study conducted by Liang et al. (22) recently reported that, in Italy, higher levels of parental stress due to the COVID-19 pandemic predicted more severe anxiety and depression in their adolescent children. After the stay-at-home orders were implemented in the United States, a transactional association between parent and child took place, where parents' reported anxiety and depressive symptoms predicted adolescents' reports of the same symptoms 1 month later (23). Moreover, previous studies demonstrate that the COVID-19 pandemic increased mothers' perceived vulnerability to the virus (6) and led to an increase in maternal anxiety and depression (24, 25), suggesting the need to pay more attention to the potential impact of maternal mental health problems on adolescents. According to previous epidemiological studies, PTSD symptoms, negative affect, anxiety, and depression are considered important indicators of youth mental health during the COVID-19 pandemic (2, 3). In the present study, we focused on the relationship between maternal anxiety during the COVID-19 pandemic and adolescents' psychological maladjustment (i.e., PTSD symptoms, negative affect, anxiety, and depressive symptoms) and anticipated spillover effects such that maternal anxiety would be associated with adolescents' mental health.

Self-compassion in adolescents may buffer the impact of maternal anxiety on adolescents' mental health during the COVID-19 pandemic. Self-compassion, as defined by Neff (26), entails three basic components: (1) self-kindness, an attitude for extending kindness and understanding to oneself rather than harsh judgment and self-criticism; (2) common humanity, seeing one's experiences as part of the larger human experience rather than separated and isolated; and (3) mindfulness, a capacity for holding one's painful thoughts and feelings in balanced awareness rather than over-identifying with them. A meta-analysis of 19 studies conducted with adolescents found a large effect size for a negative relationship between self-compassion and psychological distress (indexed by anxiety, depression, and stress) (27), which indicated a protective effect of self-compassion for adolescents when met with personal challenges or difficult life circumstances. Evidence from empirical studies also supports the protective role of self-compassion in adolescents (28, 29). For example, Latinen et al. (28) found that, among high school students, self-compassion could alleviate the relationship between academic difficulties and depressive symptoms. A longitudinal study found that self-compassion served as a protective factor in preventing the transition from suicidal ideation to suicide attempt in Chinese adolescents (29).

To our knowledge, few studies directly examine the protective role of self-compassion among adolescents in the context of COVID-19. There is some evidence of the protective role of self-compassion that appeared among adults during the COVID-19 pandemic (30, 31). For instance, Lau et al. (30) found that self-compassion could buffer the impact of COVID-19 on Hong Kong residents' psychological distress during the peak of the local outbreak of the virus. Additionally, a 14-day mobile self-compassion intervention was found to have the ability to reduce stress and help participants maintain healthy eating habits during the COVID-19 pandemic (31). Theoretically, self-compassion may protect adolescents from maternal COVID-19 anxiety through three mechanisms. First, preventing negative self-talk, and promoting self-kindness may make it easier for adolescents to maintain the balanced awareness of other's emotions (i.e., maternal anxiety) and their own emotions (32), thus preventing the negative emotional transaction from mother to child. Second, through common humanity, the feeling of connection (33) allows adolescents to realize that they may not be the only ones facing at mother's anxiety during the COVID-19 pandemic. This realization may reduce their psychological maladjustment. Third, mindfulness may foster a broadened perspective toward stressful situations (i.e., maternal anxiety caused by COVID-19), allowing for psychological distance between these maternal negative emotions and adolescents themselves (29). Thus, mindfulness may also work to aid adolescents in their adjustment to changing environments due to maternal anxiety about the COVID-19 pandemic.

In summary, the aim of this study was to examine the impact of maternal anxiety symptoms on adolescents' mental health (i.e., PTSD symptoms, negative affect, anxiety, and depression symptoms) in the context of COVID-19 and explore the moderating role of adolescents' self-compassion on the relationship between maternal anxiety symptoms and adolescents' mental health. Two hypotheses were proposed:

Hypothesis 1. Maternal anxiety during the COVID-19 pandemic is expected to be positively associated with adolescents' psychological maladjustment.
Hypothesis 2. Self-compassion is expected to serve as a moderator in the relationship between maternal anxiety and adolescents' psychological maladjustment. Specifically, adolescents with higher levels of self-compassion would experience less of an impact of maternal anxiety on their mental health during the COVID-19 pandemic.

## Methods

### Participants

Adolescents in this study were recruited from three public schools in Zhengzhou, Henan province. The city is located in central China with a population of approximately 10.35 million. After obtaining relevant informed consent, the final sample included 5,720 adolescents (*M*_age_ = 11.60, ranging from 10 to 17 years, *SD*_age_ = 1.36, 48.9% girls) and their mothers (*M*_age_ = 39.75, *SD*_age_ = 4.21). The majority of the adolescents came from families of lower-middle to middle socioeconomic status. This distribution was representative of the general population in Zhengzhou based on data from the Bureau of Statistics of Zhengzhou (34). Table 1 presents demographic information reported by adolescents and their mothers in the current study.

**Table 1:** Demographic information of the sample (*N* = 5720).

**Categories**	***M* (*SD*)%**
**Adolescent characteristics**	
Age (year)	11.60 (1.36)
Only child	24.0%
**Gender**	
Boys	51.1%
Girls	48.9%
**Grade**	
Primary school (grade 4–6, age range: 10–14 years)	82.3%
Middle school (grade 7–9, age range: 10–17 years)	17.7%
**Living characteristics during COVID-19(mother report)**	
**Residence during the COVID-19 pandemic**	
Low risk Middle risk	60.3% 35.7%
High risk	4.0%
**Knowledge of relatives or close friends infected by COVID-19**
Yes	0.9%
No	99.1%
**Knowledge of someone infected by COVID-19 in the same community**	
Yes	13.5%
No	86.5%
**Family Characteristics**	
**Maternal Age (year)**	39.75 (4.21)
**Maternal education**	
Middle school or below	18.0%
High school or technical secondary school	31.0%
Junior college or higher vocational college degree	26.2%
Bachelor's degree	21.7%
Master's degree or above	3.0%
**Family Income (per year)**	
¥30,000 or below (approx $4,641 or below)	16.1%
¥30,000–50,000 (approx $4,641–7,736)	19.0%
¥50,000–100,000 (approx $7,736–15,472)	28.2%
¥100,000–150,000 (approx $15,472–23,209)	16.9%
¥150,000–200,000 (approx $ 23,209–30,945)	11.4%
¥200,000–400,000 (approx $ 30,945–61,891)	6.1%
¥400,000 or above (approx $ 61,891 or above)	2.2%

### Procedure

Data for the current study were collected in April 2020 when schools in Zhengzhou remained closed. Data on adolescents and their mothers were collected through a Chinese online platform (Wenjuanxing). Due to the COVID-19 school closure, our research assistants organized online information groups to communicate with participants after access was granted by teachers and principals in local schools. The online survey was designed as a closed survey, and the potential participants could only access the survey through the links offered by research assistants in the groups. The online consent forms were sent to parents in the groups with the invitation to mothers and adolescents to participate in the current study. 96% of parents provided consent with the completed maternal survey, and 100% of adolescents with parental consent assented to participate and completed the online survey. Survey questions on demographic information were optional. All other survey items were mandatory with nonresponse options (e.g., “not applicable” or “rather not say”). The study's procedure was approved by the Research Ethics Review Board at East China Normal University.

### Measures

#### Maternal Anxiety

Mothers self-rated symptoms of anxiety during the COVID-19 pandemic utilizing the 7-item Generalized Anxiety Disorder-7 (e.g., “not being able to stop or control worrying,” “becoming easily annoyed or irritable”) (35). All the items were rated on a 4-point Likert scale, ranging from *never* to *almost every day* pertaining to the previous 2 weeks. The mean score was used as the final indicator of the severity of anxiety symptoms. Previous studies show that this questionnaire has high reliability and validity in China (36). Internal consistency reliability was 0.93 in the current study.

#### Self-Compassion

The abbreviated version of the Neff self-compassion questionnaire (37), revised by Chinese scholars (38), was used to examine adolescents' self-compassion. The 12-item scale used in the current study contains three dimensions: self-tolerance (three items, e.g., “I can accept my own shortcomings and deficiencies”), universal humanity (four items, “When I fail in important things, I always feel isolated and helpless” reverse scored), and mindfulness (five items, e.g., “When things get worse, I can understand that frustration is part of the life experience”). All items were rated on a 5-point Likert scale, from *never* to *always*. The mean score was calculated with higher scores indicating higher levels of adolescents' self-compassion. Previous studies find great reliability and validity in this scale (38) for Chinese adolescents. The Cronbach's α in the current study was 0.73.

#### PTSD Symptoms

The 17-item PTSD Checklist [PCL; (39)] was used to assess the level of adolescents' PTSD symptoms during the COVID-19 stay-at-home period. Pertaining to the previous 7 days, adolescents rated items measuring symptoms of PTSD (e.g., “I have nightmares,” “I am sad to think of what has happened”). These items were measured utilizing a 4-point Likert-scale, ranging from *no* to *always* in regards to their experience with the COVID-19 pandemic. The mean score was calculated with higher scores indicating a greater influence of the COVID-19 pandemic on the adolescents' psychological status. Previous research using the PCL revealed its strong internal consistency and good test–retest reliability (40). The Cronbach's α was 0.88 in the current study.

#### Negative Affect

Adolescents reported their feelings regarding the previous week using the revised Positive and Negative Affect Schedule for Children (PANAS-C). The original short-version scale is composed of two subscales, Positive Affect (PA) and Negative Affect (NA). Both subscales contain five items (41) evaluating the intensity of each type of emotion experienced by the respondent for a certain period of time. For this study, we simplified the questionnaire into six items in total with three items from each subscale. Those of the NA subscale include “I felt nervous,” “I felt upset,” and “I felt afraid.” All items were rated on a 7-point Likert scale (from 1 = none to 7 = completely). The original scale has been validated in Chinese adolescent samples (42). The internal consistency reliability was 0.74 in this study.

#### Anxiety and Depression

Mothers reported adolescents' emotional status utilizing the subscale (i.e., Anxious/Depressed) of the Child Behavior Checklist [CBCL; (43)]. Example items were “worry and anxiety” and “prone to crying.” All items were rated on a 5-point Likert scale, ranging from *non-conformed* to *very conformed* with the mean score determining the emotional state of the adolescents over the previous 2 weeks. This measure has been validated in Chinese adolescent samples (44). Internal consistency reliability was 0.93 in the current study.

### Statistical Analysis

The statistical analyses were conducted using SPSS version 23.0 and Mplus 8.3. Primary analyses included descriptive statistics, tests for gender and grade differences with MANOVAs, and correlation analyses.

Latent moderated structural equation models [LMS; (45)] were used to evaluate hypothesis 2. A maternal anxiety latent variable was created using the original seven items. A self-compassion latent variable was created using its three dimensions: self-tolerance, universal humanity, and mindfulness. Psychological maladjustment was indicated using adolescents' PTSD symptoms, negative affect, anxiety, and depression. Confirmatory factor analysis was first conducted to test the measurement models of three latent variables (maternal anxiety, self-compassion, and psychological maladjustment). Two steps of LMS were then used to test the moderating effect of self-compassion.

The first step estimated the structural model without the latent interaction term (Model 0). The second step estimated the structural model, including the latent interaction term (Model 1). It should be noted that the latent moderated model does not report fit indices (i.e., comparative fit index [CFI], Tucker–Lewis index [TLI], root mean square error of approximation [RMSEA]); however, the latent interaction term does not affect the fit of the measurement model (46). The log-likelihood ratio test was used to determine whether the more parsimonious Model 0 represents a significant loss in fit relative to the more complex Model 1 (46). The test equation was as follows:


$$\begin{array}{l} \text{D}=-2\left[\left(\text{log}-\text{likelihood}M0\right)--\left(\text{log}-\text{likelihood}M1\right)\right] \end{array}$$

The values of *D* are distributed approximately as χ^2^with *df* equal to the difference in free parameters between the two models. If Model 0 fits well and the log-likelihood ratio test is significant, then the researcher can conclude that Model 1 is also a well fitted model, and the moderated effect is supported.

Missing data were treated using full information maximum likelihood estimation in Mplus (47). As suggested by previous studies, all continuous variables were standardized in the model to avoid multicollinearity effects. The simple slope was applied for probing interactions with the ± 1 SD of the mean of self-compassion.

## Results

### Primary Analysis

Mean values and standard deviations of all study variables are shown in Table 2. The exploratory multivariate analysis of variance (MANOVA) test revealed a significant main effect of gender (1 = boys, 2 = girls), Wilks' λ = 0.99, *F*_(5, 5705)_ = 4.46, *p* < 0.01, partial η^2^ = 0.004, and grade (1 = primary school, 2 = middle school), Wilks' λ = 0.97, *F*_(5, 5705)_ = 31.88, *p* < 0.01, partial η^2^ = 0.03, and interaction effects between gender and grade, Wilks' λ = 0.997, *F*_(5, 5705)_ = 3.27, *p* < 0.01, partial η^2^ = 0.003.

**Table 2:** Descriptive statistics of and correlations among the study variables.

	***M*(*SD*)**	**1**	**2**	**3**	**4**	**5**	**6**
1. Gender	NA						
2. Grade	NA	0.06^**^					
3. Maternal anxiety	1.64 (0.61)	−0.03^*^	−0.01				
4. Adolescent self-compassion	3.44 (0.59)	−0.02	−0.08^**^	−0.23^**^			
5. Adolescent PTSD symptoms	1.53 (0.43)	0.02	0.08^**^	0.25^**^	−0.34^**^		
6. Adolescent negative affect	2.36 (1.26)	0.04^**^	0.16^**^	0.21^**^	−0.31^**^	0.62^**^	
7. Adolescent anxiety and depression	1.79 (0.84)	−0.02	0.02	0.43^**^	−0.33^**^	0.31^**^	0.24^**^

*Gender was dummy coded: 0 = boys, 1 = girls. Grade was dummy coded: 0 = primary school, 1 = middle school.*

* *p < 0.05,*

** *p < 0.01*.

For *gender* differences, the subsequent univariate analyses showed that, compared with boys, girls reported lower levels of self-compassion, higher levels of PTSD symptoms, and higher levels of negative affect. For *grade* differences, the univariate analyses indicated that, compared with primary school students, students in middle school reported lower levels of self-compassion, higher levels of PTSD symptoms, and higher levels of negative affect during the COVID-19 stay-at-home period. For the *gender* × *grade* group interaction, results from simple-effect analyses indicated that girls reported lower levels of self-compassion than boys in middle school, whereas there was no significant gender difference in self-compassion in primary school. Furthermore, girls reported higher levels of PTSD symptoms and negative affect than boys in middle school, whereas such gender differences were not significant in primary school.

Intercorrelations between all study variables are presented in Table 2. In short, the pattern of associations between these variables is consistent with our hypotheses. Maternal anxiety during the COVID-19 pandemic was found to be negatively associated with adolescents' self-compassion and positively associated with each of the indices of adolescents' psychological maladjustments. Moreover, adolescents' self-compassion was negatively associated with every measurement of adolescents' psychological maladjustment.

### Maternal Anxiety and Adolescents' Psychological Maladjustment: Self-Compassion as a Moderator

Results of the confirmatory factor analysis indicated that the measurement model evaluated in our primary analysis fit the data well, χ^2^ = 1987.48, *df* = 61, CFI = 0.93, TLI = 0.91, RMSEA = 0.07, SRMR = 0.06. Given the gender and grade differences found in the primary analyses, we explored the gender and grade differences in the relationship between maternal anxiety and adolescents' psychological maladjustment. The results indicate that there were no gender or grade differences found in these relationships. Thus, in the subsequent latent moderated structural equation model, adolescents' gender and grade combined with maternal educational level, annual family income, and COVID-19 related risk living area were included in the analyses as *covariates*.

The model fit indices for the latent moderated structural equation model are presented in Table 3. As evident in this table, the result indicated that Model 0 with the main effect of self-compassion fit the data well, χ^2^ = 2,659.84, *df* = 121, CFI = 0.92, TLI = 0.90, RMSEA = 0.06, SRMR = 0.06. As noted above, Model 1 included the latent interaction terms (maternal anxiety × self-compassion) on the basis of Model 0. The log-likelihood ratio test found that the fit of Model 1 (likelihood value = −88,069.21) was better than the fit of Model 0 (likelihood value = −88,092.68), *D* (*df* = 1) = 46.94, *p* < 0.001. These results indicate that the interactive effect of maternal anxiety and self-compassion on adolescents' psychological maladjustment was significant, β = −0.16, *p* < 0.01.

**Table 3:** Model fit indices and regression coefficients for the latent moderated model.

	**Model 0**	**Model 1**
χ^2^	2659.84	
*df*	121	
Log (L)	−88092.68	−88069.21
CFI	0.92	
TLI	0.90	
RMSEA	0.06	
SRMR	0.06	
**Maternal anxiety → Psychological maladjustment**	0.56^**^	0.54^**^
**Self-compassion → Psychological maladjustment**	−0.36^**^	−0.43^**^
**Maternal anxiety ×Self-compassion → Psychological maladjustment**		−0.16^**^
Gender → Psychological maladjustment	−0.002	−0.004
Grade → Psychological maladjustment	0.08^**^	0.08^**^
Maternal educational level → Psychological maladjustment	−0.04	−0.03
Family income → Psychological maladjustment	−0.02	−0.02
Living in the COVID-19 risk area → Psychological maladjustment	0.05^**^	0.05^**^

*CFI, comparative fit index; TLI, Tucker-Lewis index; RMSEA, root mean square error of approximation; SRMR, Standardized Root Mean Square Residual. Estimates are standardized coefficients.*

*Covariates are gender, grade, maternal educational level, annual family income and COVID-19 related risk living area. Gender was dummy coded: 0 = boys, 1 = girls. Grade was dummy coded: 0 = primary school, 1 = middle school.*

** *p < 0.01*.

**Figure 1:**
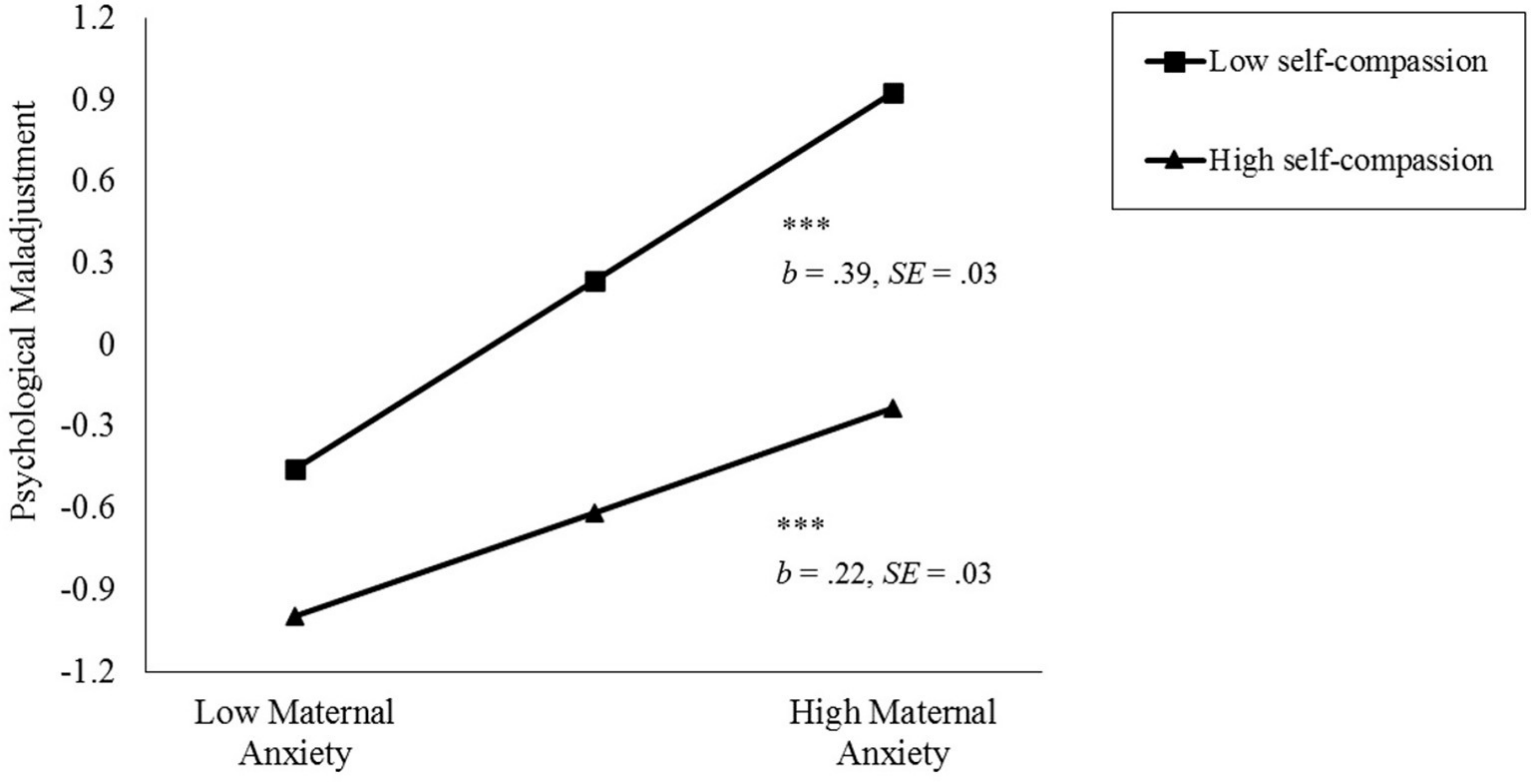
Simple slopes of the relationship between maternal anxiety and adolescents' psychological maladjustment at different levels of adolescents' self-compassion.

Furthermore, the simple slopes test indicates that, among adolescents with low self-compassion (−1 *SD*), maternal anxiety during the COVID-19 pandemic was significantly and positively associated with adolescents' psychological maladjustment (*b* = 0.39, *p* < 0.001). However, among adolescents with high self-compassion (+1 *SD*), the effect of maternal anxiety on adolescents' psychological maladjustment became weaker (*b* = 0.22, *p* < 0.001). Overall, adolescents with higher levels of self-compassion (+1 *SD*), regardless of their mothers' level of anxiety, reported fewer psychological maladjustments (i.e., below the mean level) during the COVID-19 pandemic (see Figure 1).

## Discussion

The present study investigates the potential protective factor in the transactional process of psychological distress from mothers to children in the COVID-19 pandemic context. As expected, when mothers reported more anxiety during the COVID-19 pandemic, adolescents also reported more psychological maladjustment (i.e., PTSD symptoms, negative affect, anxiety, and depression). This finding was consistent with previous research during the COVID-19 pandemic (22, 23) and provided more empirical evidence of this process in China. During the COVID-19 pandemic, parents were faced with many uncertainties and potential threats caused by the virus, such as a sudden lockdown and the possibility of infection. These concerns may raise psychological distress, such as anxiety. In addition, compared with other family members, mothers were found to face more challenges during the pandemic (19) and perceived more COVID-19 related health risks (6), which may further exacerbate their feelings of anxiety. The prolonged stay-at-home period resulted in parents and children spending significantly more time interacting with each other. Such an environment would make the direct transactional process of psychological distress (i.e., anxiety) from parent to child more likely to occur as suggested by the spillover hypothesis (21). Adolescents may develop more psychological maladjustments when their mothers experience high anxiety during the COVID-19 pandemic.

Importantly, our study found that adolescents' self-compassion was an important protective factor that buffered the association between maternal anxiety and adolescents' psychological maladjustment during the COVID-19 pandemic. Adolescents with high levels of self-compassion not only experienced a weaker impact of maternal anxiety, but also reported less psychological maladjustment regardless of their mother's emotional status. Self-compassion is considered an adaptive self-regulatory skill, especially for adolescents (28). When exposed to maternal anxiety, adolescents with higher levels of self-compassion may show more understanding of the negative emotions they witnessed in their mothers (i.e., “mother is facing more stress during the COVID-19, so she is in a bad mood”) and hold fewer harsh judgments and more self-kindness for themselves also. Mindfulness may increase the psychological distance between maternal anxiety and adolescents themselves, reducing the impact of this negative emotion on them. Besides this, prior studies find that the COVID-19 outbreak has altered adolescents' social patterns with families and friends evidenced by increased family conflict but strengthened and more selective peer relationships (48). Positive online communication with peers may increase common humanity allowing adolescents to maintain self-compassion (e.g., communicating online with friends about their mothers' anxiety and emotional state during the COVID-19 pandemic). As a result, the impact of maternal anxiety during the COVID-19 pandemic on children may be lessened.

Moreover, our findings suggest that *lower* levels of self-compassion may be a potential *risk* factor for adolescents' mental health during the COVID-19 pandemic. As seen in Figure 1, when mothers reported higher levels of anxiety symptoms, adolescents with lower levels of self-compassion (−1 SD) exhibited the highest score of psychological maladjustment as compared with other situations. Lower levels of self-compassion may indicate that these adolescents process stressful events in a maladaptive way (e.g., self-judgment, isolation, and over-identifying with distress) and, thus, experience harsher negative consequences of maternal anxiety during the COVID-19 pandemic.

It is worth discussing how adolescents in families with highly anxious mothers maintained their self-compassion during the COVID-19 pandemic. Two possible explanations were raised. First, maternal anxiety during the COVID-19 may differ from mothers' real interactions with their children. As suggested by the research of Dollberg et al. (49), mothers' mentalization skills (i.e., the ability to recognize child's mental and emotional needs) (50) during the COVID-19 pandemic were unrelated to maternal anxiety symptoms. Additionally, children were found to have a decreased risk of behavioral problems if their mothers had stronger mentalization skills (49). In this regard, some anxious mothers may wish to provide a safe and secure family environment in the unpredictable COVID-19 period for their children. They may conceal or regulate their feelings when interacting with children, which may promote self-compassion in adolescents during the COVID-19 pandemic. A second possible explanation is that an external factor, such as the setting of online school curricula rather than the family environment, may have a positive effect on the development of adolescents' self-compassion during the COVID-19 pandemic. For instance, the *School's Out, But Class's On* project launched by the Chinese Ministry of Education considered students' mental health education as greatly important during the pandemic (51). Adolescents may have opportunities to learn how to promote self-kindness and mindfulness in dealing with their negative emotions during the COVID-19 school closure period.

Finally, gender and grade differences existed in adolescents' self-compassion and psychological maladjustment with middle school girls reporting lower levels of self-compassion and higher levels of psychological maladjustment (i.e., PTSD symptoms and negative affect) than primary school girls or boys of any grade during the COVID-19 pandemic. These findings of the current study were not only consistent with previous studies (52, 53) but built on them to consider the COVID-19 context. In line with previous studies prior to the COVID-19 pandemic, older female adolescents were found to be more vulnerable to adverse conditions such as family conflict (54), showed less self-compassion (52), and experienced more psychological distress, such as anxiety, depression, and stress (53) compared with their counterparts. Furthermore, studies during the COVID-19 pandemic also reveal that older children experienced more psychological distress due to COVID-19's daily impacts compared with younger children (55). As self-compassion was examined as the protective factor buffering the impact of stressors in the current and in previous studies (30), the present study's findings suggest that more attention is needed on older female adolescents' mental health during the COVID-19 pandemic. In addition, the need to investigate how to aid older female adolescents in developing more self-compassion during the pandemic is evident from the current study's findings.

### Limitations and Future Directions

The present study offers evidence of the protective role of self-compassion in the relationship between maternal anxiety and adolescents' psychological maladjustment in the context of the COVID-19 pandemic. Several limitations should be noted in the current study along with suggestions for future research. First, the cross-sectional design precluded our ability to make causal inferences regarding the relationship between maternal anxiety and adolescents' mental health. Alternative explanations must be considered. For instance, bidirectional relationships between maternal anxiety and adolescents' mental health may be found in the family system during the COVID-19 pandemic. It is also possible that a genetic link exists between mothers and children (54), impacting the results of this study as pre-COVID-19 anxiety assessments were not given. Although adolescents' reported self-compassion was found to buffer the relationship between maternal anxiety and adolescents' psychological maladjustment, it is important to recognize these notable limitations.

Second, several measurement issues should be considered. Although the current study used a multiple-informant approach to improve the validity of adolescents' psychological maladjustment, mothers with especially high levels of anxiety symptoms may have been more likely to report anxiety and depression symptoms in their children. This may have exaggerated the results on the psychological effects of maternal anxiety on children. Moreover, the present study only investigated the negative aspects of mental health outcomes. Considering the dual-factor model of mental health (56), future studies should further explore the possible role of self-compassion on the positive aspects of mental health (e.g., life satisfaction) during the COVID-19 pandemic.

Third, it is also important to contextualize the current findings as our results may not be generalizable to other samples of individuals who were exposed to more stressful conditions. In more stressful settings, such as the “red zone” in Italy, mothers are more likely to be aware of the life-threatening risks of COVID-19 (25), and adolescents may fail to develop a certain level of self-compassion, a potential protective factor during a stressful event.

Finally, it is worthy for future studies to investigate the two possible explanations we raised to explain how adolescents developed self-compassion with their anxious mothers. Conducting qualitative studies or directly testing for the two hypothetical explanations proposed in the current study through quantitative research would provide valuable information on the possible mechanisms at play between maternal anxiety and adolescent mental health.

### Implications

The present study highlighted the protective role of adolescents' self-compassion when faced with maternal anxiety during the COVID-19 pandemic. The findings have practical implications for families, schools, and societies at large. For instance, parents should recognize their negative emotional state may have a transactional impact on their children in the family system. Thus, more emotional awareness and emotional regulation should be practiced by parents during the COVID-19 pandemic. Second, self-compassion and mindfulness training proved to be effective for adolescents before and during the COVID-19 pandemic (57, 58). School counselors and therapists could implement the self-compassion intervention to enhance adolescents' well being. Governments and institutions should also offer the public information and tools to develop adolescents' self-compassion during the pandemic to improve their mental health.

## Data Availability Statement

The raw data supporting the conclusions of this article will be made available by the authors, without undue reservation.

## Ethics Statement

The studies involving human participants were reviewed and approved by Research Ethics Review Board at East China Normal University. The participants provided their informed consent via the online link to participate in this study.

## Author Contributions

TZ, XB, and JL conceptualized the study, developed methods, ethical documentation, and study materials. XB recruited participants and collected data. TZ drafted the manuscript, with KZ's help on the Method section and YL's help on the Results section. SZ, XB, HZ, JL, and JF conducted the proofreading. JL funded the current project and supervised the whole manuscript wirting process. All authors contributed to the article and approved the submitted version.

## Funding

This work was supported by the Program of Philosophy and Social Science in Henan Province (2021BJY043), the Research Project of Shanghai Science and Technology Commission (20dz2260300), the Research Funds for Shanghai Health Commission for Academic Leader Training (GWV-10.2-XD30), and the Research Funds for Changning District Health Committee of Medical Specialty (20192002).

## Conflict of Interest

The authors declare that the research was conducted in the absence of any commercial or financial relationships that could be construed as a potential conflict of interest.

## Publisher's Note

All claims expressed in this article are solely those of the authors and do not necessarily represent those of their affiliated organizations, or those of the publisher, the editors and the reviewers. Any product that may be evaluated in this article, or claim that may be made by its manufacturer, is not guaranteed or endorsed by the publisher.
